# Development of non-viral, ligand-dependent, EPHB4-specific chimeric antigen receptor T cells for treatment of rhabdomyosarcoma

**DOI:** 10.1016/j.omto.2021.03.001

**Published:** 2021-03-05

**Authors:** Hiroshi Kubo, Shigeki Yagyu, Kayoko Nakamura, Kumiko Yamashima, Akimasa Tomida, Ken Kikuchi, Tomoko Iehara, Yozo Nakazawa, Hajime Hosoi

**Affiliations:** 1 Department of Pediatrics, Graduate School of Medicine, Kyoto Prefectural University of Medicine, Kyoto 602-8566, Japan; 2 Department of Pediatrics, Shinshu University School of Medicine, Matsumoto, Japan; 3 Institute for Biomedical Sciences, Interdisciplinary Cluster for Cutting Edge Research, Shinshu University, Matsumoto, Japan

**Keywords:** EPHB4, chimeric antigen receptor, CAR-T cell therapy, rhabdomyosarcoma, *piggyBac* transposon, stem cell memory-like T cells

## Abstract

Ephrin type-B receptor 4 (EPHB4), expressed in tumors including rhabdomyosarcoma, is a suitable target for chimeric antigen receptor (CAR)-T cells. Ligand-independent activation of EPHB4 causes cell proliferation and malignant transformation in rhabdomyosarcoma, whereas ligand-dependent stimulation of EPHB4 induces apoptosis in rhabdomyosarcoma. Therefore, we hypothesized that ligand-based, EPHB4-specific CAR-T cells may kill rhabdomyosarcoma cells without stimulating downstream cell proliferation mechanisms. We developed novel CAR-T cells by targeting EPHB4 via EPHRIN B2, a natural ligand of EPHB4. The generation of EPHB4-CAR-T cells via *piggyBac* (PB) transposon-based gene transfer resulted in sufficient T cell expansion and CAR positivity (78.5% ± 5.9%). PB-EPHB4-CAR-T cells displayed a dominant stem cell memory fraction (59.4% ± 7.2%) as well as low PD-1 expression (0.60% ± 0.21%) after 14 days of expansion. The PB-EPHB4-CAR-T cells inhibited EPHB4-positive tumor cells without activating cell proliferation downstream of EPHB4, even after multiple tumor re-challenges and suppressed tumor growth in xenograft-bearing mice. Therefore, PB-EPHB4-CAR-T cells possess a memory-rich fraction without early T cell exhaustion and show potential as promising therapeutic agents for treating rhabdomyosarcoma and other EPHB4-positive tumors.

## Graphical Abstract

**Figure:**
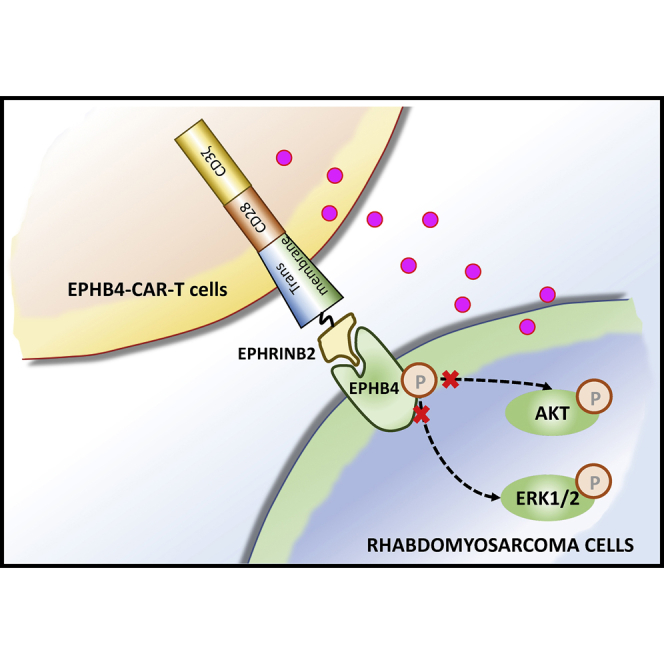

## Teaser

*piggyBac* transposon-mediated CAR-T cell therapy targeting EPHB4 exhibited sufficient CAR positivity, stable CAR expression, a favorable phenotype, and strong antitumor efficacy against EPHB4-positive tumors and would be promising for the treatment of malignant rhabdomyosarcomas.

## Introduction

The potential of using chimeric antigen receptor T (CAR-T) cells to treat blood cancers, including chemo-refractory or relapsed leukemias, has been demonstrated.^1^, ^2^, ^3^, ^4^ However, efforts to target solid tumors using CAR-T cells have been unsuccessful.^5^, ^6^, ^7^, ^8^ There might be various factors underlying the low efficacy of CAR-T cell-based therapy against solid tumors, such as the following: (1) low levels of unique tumor-associated antigens (TAAs) that are exclusively expressed in tumor cells; (2) insufficient expansion of CAR-T cells *in vivo*; (3) insufficient tumor penetration by CAR-T cells; (4) escape from immune surveillance owing to reduced tumor antigen expression; and (5) immunosuppression owing to a unique tumor microenvironment.^9^, ^10^, ^11^ An artificial single-chain fragment variable (scFv) or a specific binding domain, such as a specific ligand of the target molecule, was utilized, and the CAR molecule was variously modified to specifically recognize TAAs.

Ephrin type-B receptor 4 (EPHB4), a member of the largest family of receptor tyrosine kinases (RTKs), is highly expressed in various tumors, such as lung cancer,^12^ colorectal cancer,^13^ breast cancer,^14^ esophageal cancer,^15^ melanoma,^16^ and malignant soft tissue sarcoma, including rhabdomyosarcoma (RMS).^17^ Ephrin receptors bind to Eph receptor-interacting ligands, which are transmembrane ligands located at sites of cell-to-cell contact.^18^^,^^19^ EPHB4 is mainly expressed in the developmental phase of three-dimensionally organized human tissue structures such as vascular formations and tissue border formations.^20^^,^^21^ Stimulation of EPHB4 by its ligand, EPHRIN B2, generates a bidirectional signaling cascade in both the cell expressing the receptor and the cell presenting the ligand.^22^ The EPHB4-EPHRIN B2 axis regulates various cellular functions such as cell migration, repulsion, and adhesion.^21^^,^^22^ Interestingly, ligand-independent activation of EPHB4 in cancer cells results in cell proliferation and malignant transformation. By contrast, ligand-dependent activation of EPHB4 induces apoptosis in some tumors.^23^ EPHRIN B2-dependent stimulation of EPHB4 activates Crkl downstream of EPHB4 signaling and induces apoptosis, especially in RMS.^24^ Based on these observations, we hypothesized that EPHB4 may be utilized as a potent target for the recognition of RMS cells. Furthermore, EPHRIN B2 could be applied to the antigen-recognition domain of the CAR construct to harness EPHB4-expressing tumor cells to genetically modify CAR-T cells that lack the proliferative tendency of tumor cells via ligand-antigen interaction. In the present study, we developed *piggyBac* (PB)-mediated CAR-T cells by redirecting EPHB4 (EPHB4-CAR-T cells) and thereby demonstrated the properties and antitumor efficacy of these cells against EPHB4-positive solid tumors *in vitro* and *in vivo*.

## Results

### EPHB4 expression in human tumor cells

To determine whether EPHB4 is a suitable target for EPHB4-CAR-T cells, we investigated the expression of EPHB4 in tumor cells. EPHB4 was expressed in several tumor cell lines including RMS at various levels (Figure S1A). Next, we used immunohistochemistry to examine EPHB4 expression in various tumors as well as in corresponding normal tissues and found that EPHB4 was expressed in some tumor tissues including invasive breast cancer, whereas it was rarely expressed in normal tissues, with the exception of placental tissue (Figure S1B).

### Generating EPHB4-CAR-T cells by PB-mediated gene transfer

**Figure 1:**
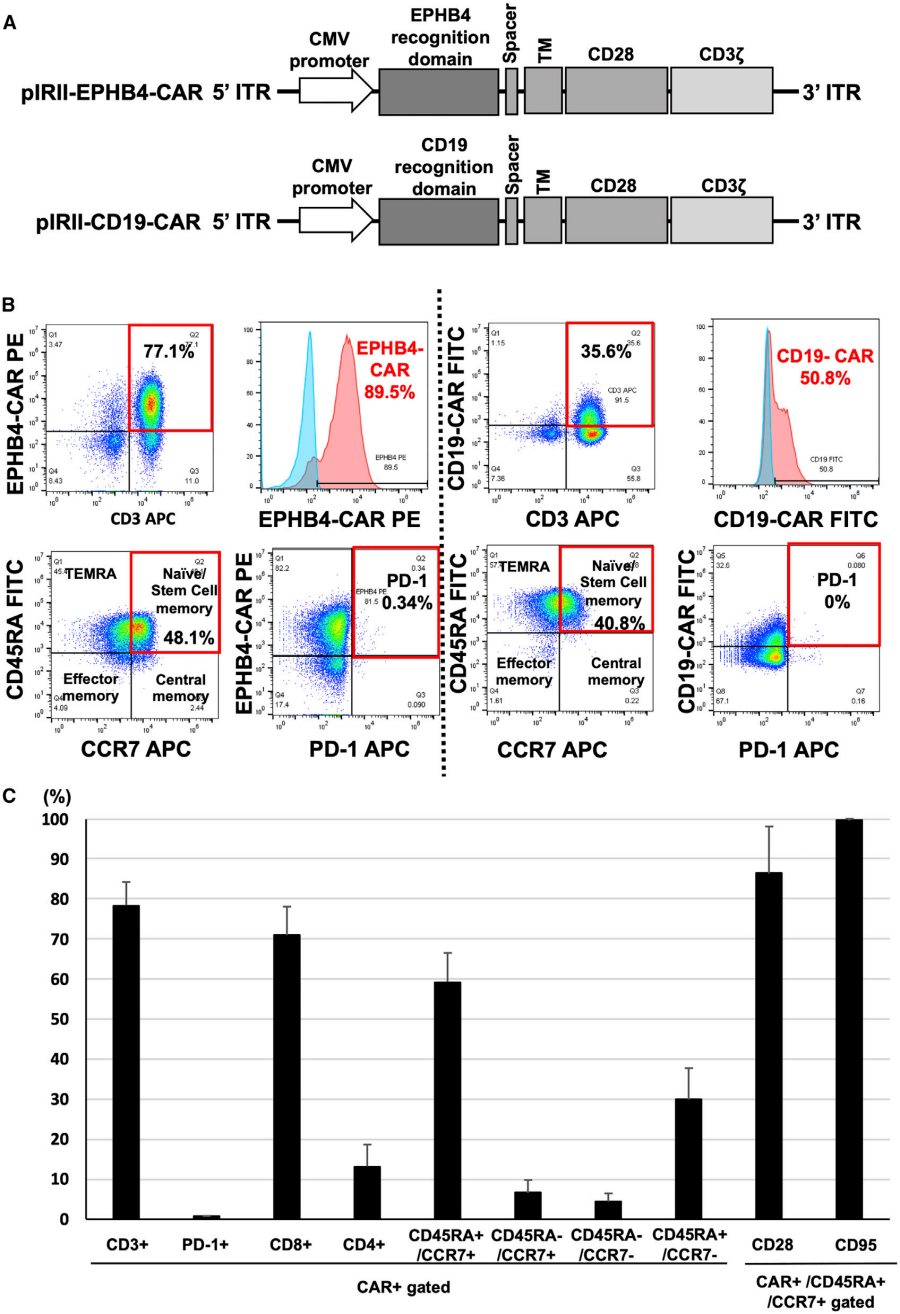
Production and expansion of EPHB4-CAR-T cells by *piggyBac*-mediated gene transfer and phenotypic analysis (A) Schematic of the EPHB4-CAR construct and CD19-CAR construct. (B) EPHB4-CAR expression, phenotypes, and expression of exhaustion markers on CAR-T cells were assessed via flow cytometry (left). CD19-CAR expression, phenotypes, and expression of exhaustion markers on CAR-T cells were assessed via flow cytometry (right). (C) The phenotype and exhaustion marker of EPHB4-CAR-T cells are represented by the mean ± SD of 5 different donors.

We generated a PB transposon-mediated plasmid encoding the extracellular portion of EPHRIN B2 fused to a second-generation CAR construct (pIRII-EPHB4-CAR-28z) and a CD19 construct as a control (pIRII-CD19-CAR-28z) (Figure 1A). A total of 4 × 10^7^ peripheral blood mononuclear cells (PBMCs) from a healthy donor were electroporated with pIRII-EPHB4-CAR-28z or pIRII-CD19-CAR-28z and PB transposase plasmids to produce EPHB4-CAR-T or CD19-CAR-T cells, respectively. Transfected cells were stimulated and expanded, as described (Materials and methods). After 14 days of expansion, we successfully generated EPHB4-CAR-T cells with 78.5% ± 5.9% CAR positivity (Figures 1B and 1C). We then determined the phenotype and the expression of the exhaustion marker on CAR-T cells via flow cytometry. These CAR-T cells had a skewed CD8-positive population (71.0% ± 7.1%) compared to the CD4-positive (13.2% ± 5.5%) population (Figure 1C). Notably, the EPHB4-CAR-T cells predominantly exhibited the CD45RA^+^/CCR7^+^ fraction (59.4% ± 7.2%) (Figure 1C), and these trends were also confirmed in the CD4-positive and CD8-positive populations, separately (Figure S2A). Moreover, this CD45RA^+^/CCR7^+^ fraction faction also exhibited high positivity for CD95 (99.7% ± 0.44%) and CD28 (86.1% ± 11.8%) populations, indicating that the T stem cell memory fraction was enriched (Figure 1C).^25^ This fraction was associated with profound and durable antitumor efficacy,^26^, ^27^, ^28^ and whereas the exhaustion marker Tim3 was considerably expressed on T cells (53.4% ± 17.2%), the expressions of PD-1 (0.60% ± 0.21%) and LAG3 (19.0% ± 3.7%) were modest, even after robust cellular expansion (Figures 1B, 1C, and S2B), indicating potential persistent proliferation following antigen stimulation.

### EPHB4-CAR-T cells displayed robust and sustained antitumor activity against EPHB4-positive tumors *in vitro*

**Figure 2:**
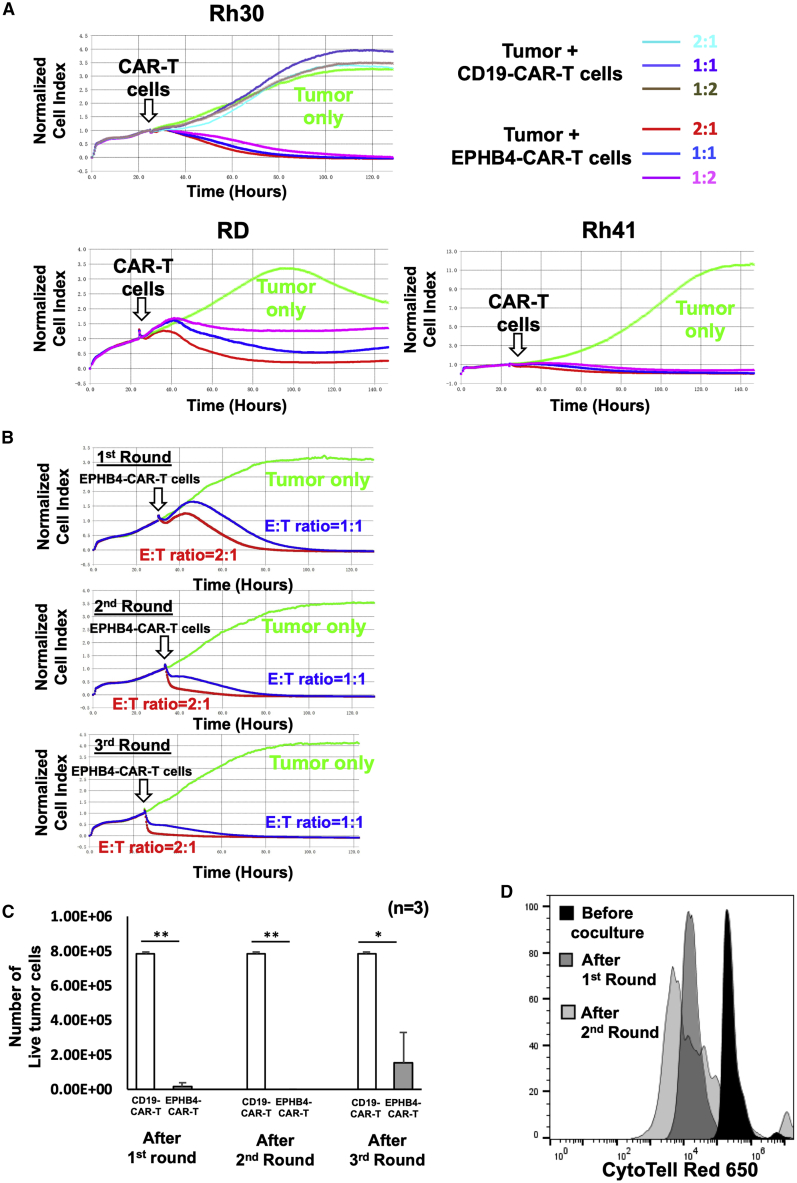
EPHB4-CAR-T cells demonstrated rapid and sustained antitumor activity against EPHB4-positive tumors EPHB4-CAR-T cells, generated by our original method, were co-cultured with EPHB4-positive tumor cells at effector:target (E:T) ratios, and normalized cell index was monitored using a xCELLigence real-time cell analysis. (A) Rh30, Rh41, and RD were co-cultured with EPHB4-CAR-T cells at E:T ratios of 2:1, 1:1, and 1:2 (B and C) Serial tumor rechallenge assay; tumor cell growth was monitored via a xCELLigence real-time cell analysis (B), and the number of live tumor cells 72 h after co-culture with CD19-CAR-T cells or EPHB4-CAR-T cells in each round was measured via flow cytometry (C). Mean ± SD from 3 different experiments are shown. ∗∗p < 0.01. ∗p < 0.05. (D) Cell division of EPHB4-CAR-T cells upon repeated Rh30 cells stimulation.

We evaluated the ability of EPHB4-CAR-T cells to kill tumor cells in co-culture assays. EPHB4-CAR-T or CD19-CAR-T cells were co-cultured with the EPHB4-positive RMS cell line (Rh30) at various effector-to-target (E:T) ratios, such as 2:1, 1:1, or 1:2, and the growth of tumor cells was monitored for over 150 h. The antitumor effects of EPHB4-CAR-T cells on other RMS cell lines, such as Rh41, RD, osteosarcoma cell line U2OS, and the breast cancer cell line BT549, were also assessed. EPHB4-CAR-T cells demonstrated potent and sustained killing activity against tumor cells, including RMS, osteosarcoma, and triple-negative breast cancer cell lines (Figures 2A and S3A). We also used a serial tumor challenge assay to evaluate the persistence of the antitumor activity of EPHB4-CAR-T cells. EPHB4-CAR-T cells displayed strong sustained killing activity against Rh30 tumor cells (Figures 2B, 2C, and S3B) and continuous proliferation (Figure 2D) even following multiple tumor rechallenge, which indicated that antitumor efficacy was not reduced due to immune evasion, a condition often observed in virally engineered CAR-T cells.^29^

### *PAX3-FOXO1* (P3F) fusion gene did not impair the antitumor activity of the EPHB4-CAR-T cells

**Figure 3:**
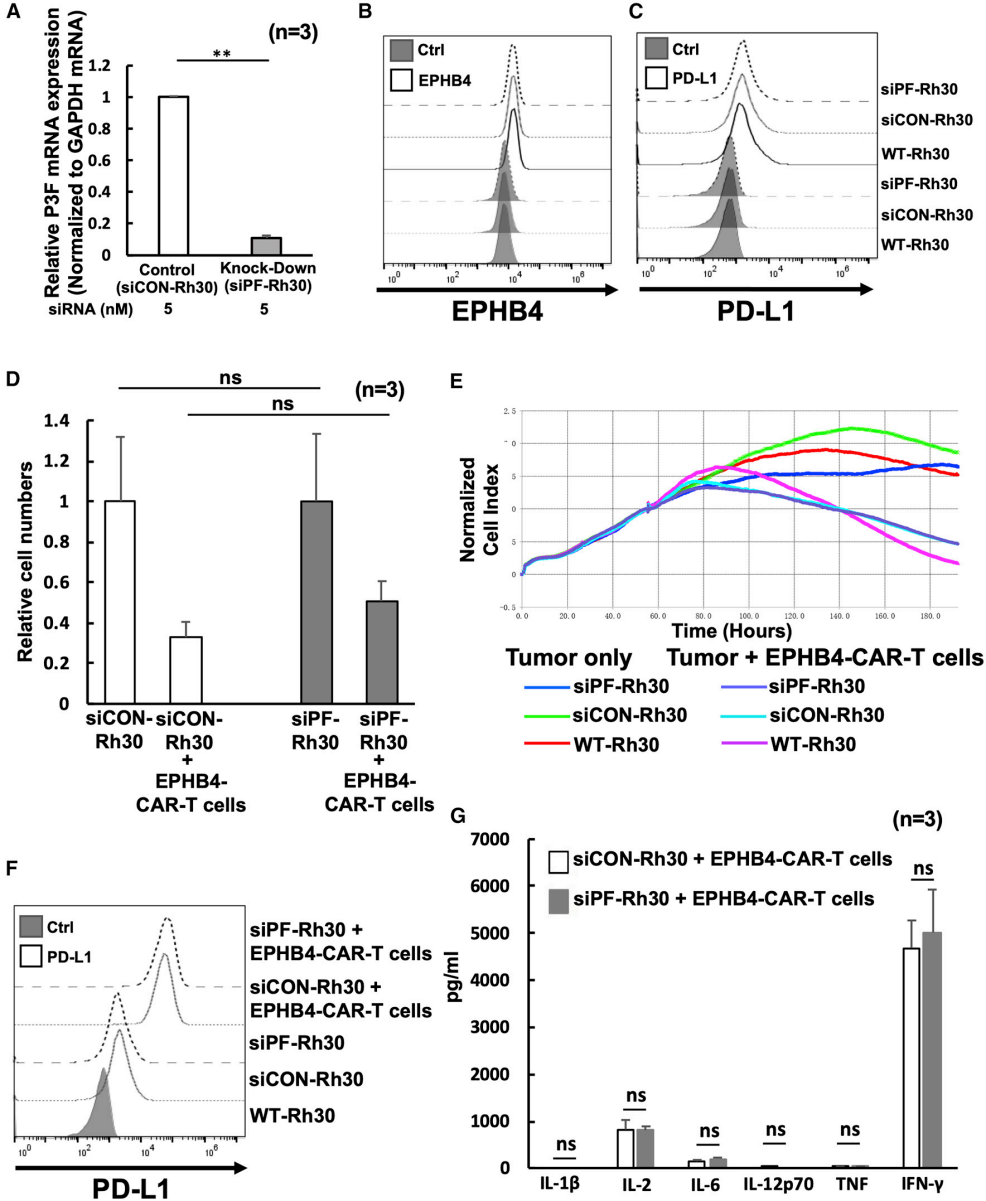
*PAX3-FOXO1* (P3F) fusion gene did not affect the antitumor efficacy of EPHB4-CAR-T cells (A) After transfection with siRNAs against P3F (siPF) into Rh30 cells for 24 h, the knocked-down efficacy of siPF against P3F was assessed by quantitative reverse-transcription polymerase chain reaction (qRT-PCR). ∗∗p < 0.01. (B and C) P3F did not affect EPHB4 (B), and PD-L1 was assessed by flow cytometry (C). (D) Comparison of the antitumor effect in siCON-Rh30 and siPF-Rh30 assessed by flow cytometry. The antitumor effect of the EPHB4-CAR-T cells on the Rh30 cells was evaluated according to the ratio of survival of the Rh30 cells alone to the survival of the Rh30 cells co-cultured with EPHB4-CAR-T cells. Mean ± SD from 3 different experiments are shown. N = not significant. (E) Cell growth of siCON- and siPF-Rh30 co-cultured with EPHB4-CAR-T cells was acquired by a xCELLigence real-time cell analysis. (F) PD-L1 expression between siCON-Rh30 and siPF-Rh30 treated with EPHB4-CAR-T cells was evaluated via flow cytometry. (G) The level of cytokines in the co-culture supernatant siCON-Rh30 or siPF-Rh30 with EPHB4-CAR-T cells for 24 h.

The P3F fusion gene undergoes a signature genetic change in a majority of alveolar RMS (ARMS) cases. In ARMS, P3F promotes malignant phenotypes such as those characterized by proliferation, motility, and suppression of differentiation.^30^^,^^31^ Because the P3F fusion gene also exerts an immunomodulatory effect that inhibits the immune activity of surrounding immune cells,^32^ we hypothesized that P3F translocation-positive RMS may circumvent the antitumor activity of CAR-T cells. To evaluate the effect of the P3F fusion gene on the function of EPHB4-CAR-T cells, we knocked down the expression of P3F in Rh30 and Rh41 cells by RNA interference (RNAi) and assessed the antitumor efficacy of EPHB4-CAR-T cells on wild-type (WT), small interfering RNA (siRNA) control (siCON)-transfected, or P3F knocked-down siRNA-transfected (siPF) Rh30 and Rh41 cells. Results indicated that the expression of P3F in siPF-Rh30 and siPF-Rh41 cells was significantly reduced (Figures 3A and S4A). However, P3F knock-down did not affect the expression of EPHB4 (Figures 3B and S4B) or PD-L1 (Figures 3C and S4C) in translocation-positive RMS cells. Although a previous study demonstrated that siPF inhibited growth and induced differentiation of Rh30 cells,^31^ we did not observe additional anti-tumor efficacy in siPF-RMS cells, which were co-cultured with EPHB4-CAR-T cells (Figures 3D, 3E, and S4D). PD-L1 expression was dramatically increased in translocation-positive RMS cells, which were co-cultured with EPHB4-CAR-T cells, indicating that immune evasion in translocation-positive RMS cells was upregulated (Figures 3F and S4E). We also evaluated the production of inflammatory cytokines in the supernatant in response to co-culture with tumor cells. Although there was a robust secretion of cytokines including interferon (IFN)-γ, there was no significant difference between the supernatants of siCON-Rh30 and that of siPF-Rh30 (Figure 3G). These data indicated that although the immunomodulatory function of P3F on translocation-positive RMS cells was evident, P3F did not affect the killing efficacy of EPHB4-CAR-T cells.

### EPHRIN B2-mediated EPHB4 activation by EPHB4-CAR-T cells did not induce RMS cell proliferation

Because the antigen-recognition site of EPHB4-CAR utilizes the natural ligand of EPHB4 (EPHRIN B2), EPHB4-CAR-T cells not only bind to EPHB4 but also stimulate it, leading to downstream activation of EPHB4 via ligand-receptor interaction. To investigate the dynamics of tumor cells resulting from ligand-dependent activation of EPHB4, EPHB4-positive Rh30 cells were co-cultured with human recombinant EPHRIN B2, and the activation of downstream signaling of EPHB4, Akt, and Erk1/2, which are associated with the cellular proliferation machinery of RMS, was evaluated. Stimulation of EPHB4 by its natural ligand EPHRIN B2 in RMS cells did not induce the phosphorylation of Akt or Erk1/2, and even further, the amount of phospho-Akt was reduced in Rh30 cells treated with EPHRIN B2 (Figure S5A). In contrast to a previous report,^24^ phospho-Crkl was not upregulated by EPHRIN B2 treatment. Nevertheless, EPHRIN B2 interaction with EPHB4 did not activate downstream EPHB4 signaling, which is involved in the cellular proliferation machinery of RMS cells. We cultured Rh30 cells on a 24-well plate pre-coated with human EPHRIN B2-Fc chimera protein to stimulate EPHB4 on RMS cells via ligand-receptor interaction. Proliferation of Rh30 cells was suppressed by EPHRIN B2-Fc stimulation, suggesting that the interaction of EPHRIN B2-Fc/EPHB4 on RMS cells likely reduced cell proliferation rather than stimulate it (Figure S5B).

### EPHB4-CAR-T cells had robust antitumor activity against EPHB4-positive tumors *in vivo*

**Figure 4:**
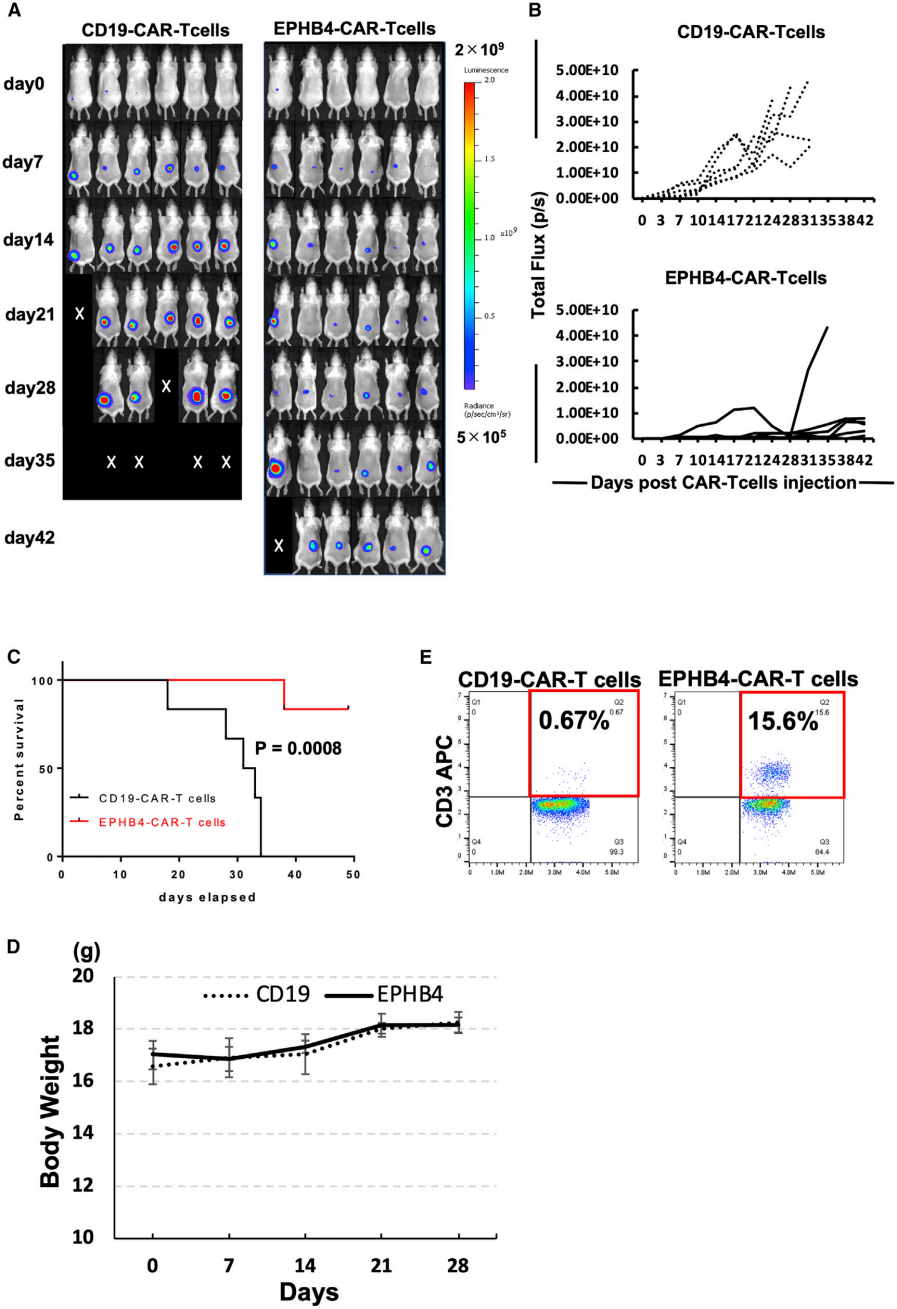
EPHB4-CAR-T cells debulked rhabdomyosarcoma tumors in a murine xenograft model We injected 2 × 10^6^ Rh30-Luc cells into the dorsal area of SCID beige mice. 7 days later, 10 × 10^6^ EPHB4-CAR-T cells or CD19-CAR-T cells (the expression of each CAR molecule was around 80%) were injected into the tail vein of each mouse. (A) Representative bioluminescent images of tumor growth over time in individual mice. (B) Kinetics of Rh30 xenograft progression in individual mice that had received either the CD19 (control) or EPHB4 CAR-T cells by IVIS imaging. (C) Survival analysis of EPHB4-CAR-T cell-treated mice and CD19-CAR-T cell-treated mice (CD19: N = 6; EPHB4: N = 6). (D) Mice treated with EPHB4-CAR-T cells did not show weight loss. (E) Human T cells in peripheral blood of mice on day 31. Representative dot plot data were shown.

In order to verify the antitumor effect of EPHB4-CAR-T cells on mouse RMS tumor xenograft models, Rh30 cells expressing firefly luciferase (Rh30-Luc) were grafted into the dorsal areas of 4-week-old female CB17.Cg-Prkdc^scid^Lyst^bg-J^/CrlCrlj mice (severe combined immunodeficiency [SCID] beige mice). After confirmation of tumor engraftment on day 7 following inoculation, EPHB4-CAR-T or CD19-CAR-T cells were injected into the tail vein of each mouse. The growth rates of tumors treated with EPHB4-CAR-T cells decreased compared to the growth rates of tumors treated with CD19-CAR-T cells (control) (Figures 4A and 4B). The mice treated with EPHB4-CAR-T cells exhibited prolonged survival (Figure 4C) without displaying signs of toxicity including weight loss (Figure 4D), indicating the antitumor efficacy of EPHB4-CAR-T cells *in vivo*. Furthermore, human CD3-positive T cells were observed in the peripheral blood of mice treated with EPHB4-CAR-T cells on day 31, whereas human CD3-positive T cells were barely detected in mice treated with CD19-CAR-T cells (Figure 4E), signifying antigen-induced proliferation of EPHB4-CAR-T cells *in vivo*.

### EPHB4-CAR-T cells recognized and physiologically interacted with murine-EPHB4-expressing cells

**Figure 5:**
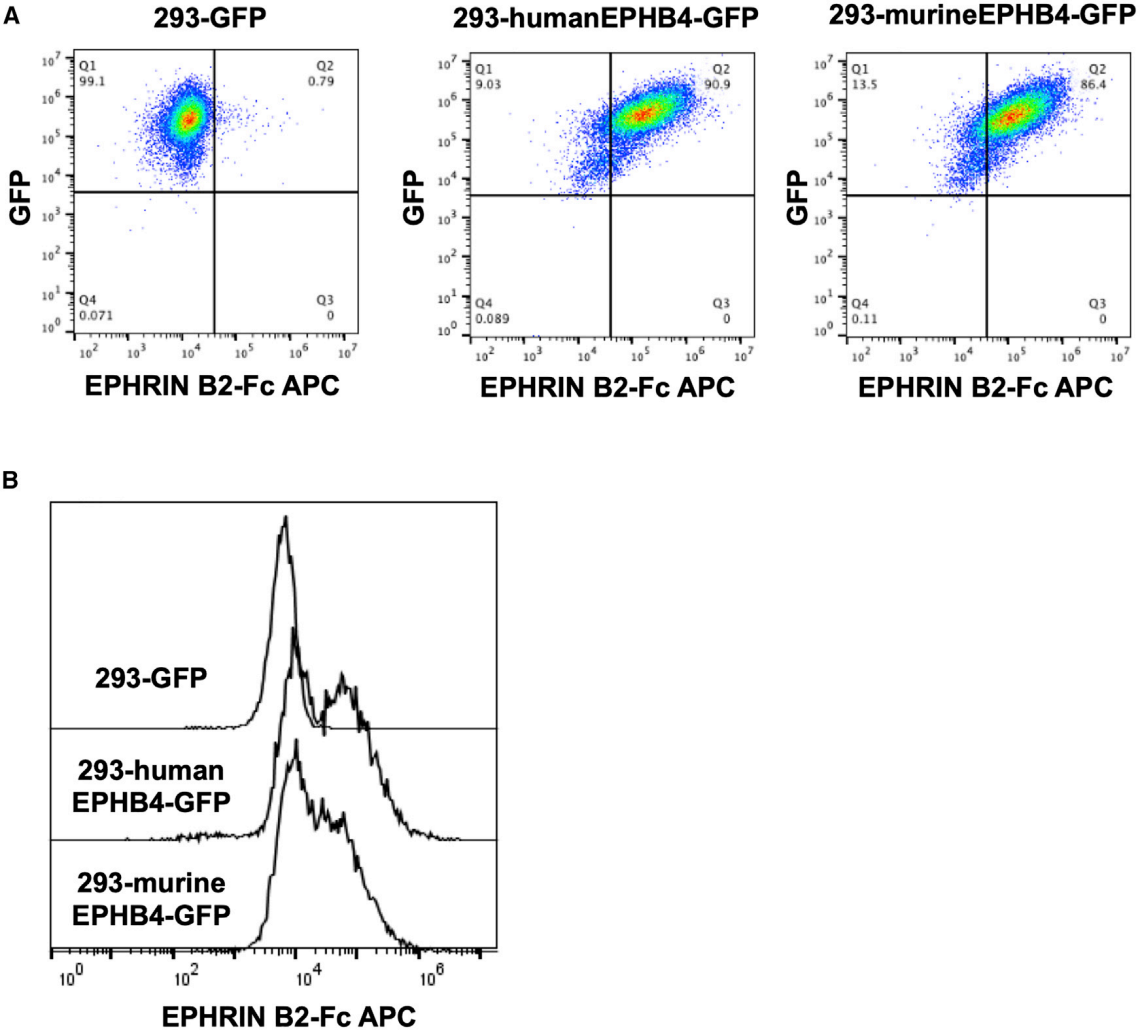
Human EPHRIN B2 protein could interact with murine-EPHB4-expressing cells (A and B) 293-GFP, 293-humanEPHB4-GFP, and 293murineEPHB4-GFP were incubated with human EPHRIN B2-Fc chimera protein, and cell-bound EPHRIN B2-Fc was detected by flow cytometry.

A thorough preclinical evaluation of toxicity, including an examination of the expression profile of the target antigen, as well as the actual binding of the antigen-recognition domain of CAR molecules to antigen-expressing tissue, should be performed before the clinical application of CAR-T cells targeting common tumor antigens in order to avoid any possibility of off-tumor toxicity. Although the immunodeficient mouse model is unsuitable for assessing immunological off-tumor/off-target toxicity due to substantial interspecies divergence between rodents and humans, the extracellular EPHB4 receptor region contains a conserved 186-amino acid N-terminal ligand-binding domain (EPHLBD) (Figure S6A), which facilitated the binding of receptors to their ephrin ligands, EPHRIN B2. The amino acid sequence of human EPHLBD shares a significantly high level of homology with murine EPHB4, with >93% overlap (Figure S6A). Similarly, the extracellular protein of EPHRIN B2 contains a conserved 137-amino acid N-terminal receptor-binding domain (Ephrin RBD) (Figure S6B), which is also required for the binding of their receptor. The amino acid sequences of human and mouse Ephrin RBDs are highly conserved, with 98.5% identity (only 1 amino acid mutation), whereas the entire extracellular portions of human and mouse EPHRIN 2 share >97% amino acid sequence identity (Figure S6B). These data indicated that human EPHRIN B2 shows potential for interacting with murine EPHB4, resulting in a cross-reaction between human EPHRIN B2 protein and murine EPHB4-expressing cells. In order to prove this, we cultured human- or murine-EPHB4-overexpressing human embryonic kidney cell line (HEK)293 cells with human EPHRIN B2-Fc chimera protein and confirmed their binding via flow cytometry. Human EPHRIN B2 protein was able to bind with both murine EPHB4 and human EPHB4 at a similar level (Figures 5A and 5B), indicating that our *in vivo* murine RMS tumor xenograft model may be utilized to ward off any possibility of off-tumor toxicity. Indeed, we did not observe any signs of adverse effects including weight loss (Figure 4E), suggesting that off-tumor toxicity induced by murine EPHB4/human EPHB4-CAR-T cell interaction was negligible.

## Discussion

We modified the extracellular domain of the EPHRIN B2 molecule as an antigen-recognition site in order to develop novel CAR-T cells that re-direct to EPHB4. Although the expression of EPHB4 in normal tissues is low,^12^^,^^33^ it is highly and ubiquitously expressed in RMS tumor tissue, including P3F translocation-positive RMS and other solid tumors.^33^, ^34^, ^35^ Therefore, we focused on the EPHB4 molecule as a favorable target for CAR-T cells. The expression levels of mRNA as well as the protein of EPHB4 are exclusively elevated in human RMS tumors, compared to those in adjacent normal skeletal muscles.^24^ Furthermore, we observed that although immunohistochemical analysis of a normal tissue panel does not reveal strong expression of EPHB4 in vital organs, it reveals weak EPHB4 expression in normal placental tissue (Figure S1B). Moreover, even though human EPHRIN B2 interacted with murine EPHB4 protein, no adverse effects were observed in the murine model, refuting safety concerns regarding EPHB4-directed therapy. These data suggested that the EPHB4 molecule was ideal for targeting by CAR-T cells and less likely to be associated with life-threatening on-target/off-tumor toxicity. Another rationale for EPHB4-targeted treatment in RMS is the correlation between EPHB4 expression and poor prognoses for patients with P3F translocation-positive RMS.^24^ Considered together, this evidence is consistent with a potential clinical relevance for targeting EPHB4 in RMS.

Most clinically investigated CAR molecules are created by fusing an antigen-binding scFv to the CAR backbone. However, we applied the extracellular domain of EPHRIN B2 to the antigen-recognition site of EPHB4-CAR for two reasons. First, EPHRIN B2, the only Ephrin ligand that interacts with EPHB4, supposedly binds EPHB4 with high affinity and shows less potential for displaying an unwanted immune response against a foreign transgene.^36^^,^^37^ Second, although EPHRIN B2-dependent interactions with EPHB4 are associated with cellular proliferation,^23^^,^^38^^,^^39^ and the blockade of EPHRIN B2-EPHB4 binding may be a therapeutic target in some tumor cells,^40^, ^41^, ^42^ EPHRIN B2-dependent interactions with EPHB4 do not promote cellular proliferation in RMS cells.^24^ Moreover, the crosstalk between EPHB4 and platelet-derived growth factor receptor β (PDGFRβ), which facilitates PDGF ligand-dependent, EPHRIN B2-independent activation of EPHB4 converging on the Akt and Erk1/2 pathways, indicated that EPHB4 and its downstream pathway may constitute a vital cellular proliferation mechanism in RMS.^24^ In the current study, we also investigated whether ligand-dependent activation of EPHB4 activates cellular proliferation and discovered that interaction between the extracellular domain of EPHRIN B2 and EPHB4 did not activate the cellular-proliferation mechanism downstream of EPHB4 signaling. Similarly, a previous study demonstrated that an EPHRIN B2-induced EPHB4 signaling cascade, mediated by the Abl-Crkl pathway, resulted in apoptosis in mammary carcinoma.^43^ In this study, the proliferation of Rh30 cells was impaired by EPHRIN B2-Fc stimulation (Figure S5B). These data indicated that the extracellular domain of EPHRIN B2 may act as an ideal antigen-recognition domain for EPHB4-CAR.

In addition to tumor proliferation, motility, and the suppression of differentiation in RMS, P3F may be associated with an immune escape mechanism.^30^^,^^31^ In our study, knockdown of *P3F* did not suppress PD-L1 expression in Rh30 cells following co-culture with EPHB4-CAR-T cells for 24 h (Figure 3F). Moreover, the EPHB4-CAR-T cells showed a very high antitumor effect against P3F-positive ARMS, as opposed to the limited immunomodulatory effect of P3F on RMS targeted by CAR-T cells. Nevertheless, further modification of EPHB4-CAR-T cells may be required in order to increase its effectiveness and safety for clinical applications. Co-expression of cytokines,^44^^,^^45^ chemokine receptor ligands,^46^ and combinations of immune checkpoint inhibitors, such as PD-1 antibodies,^47^^,^^48^ may increase the persistence and efficacy of CAR-T cells *in vivo*. Furthermore, the co-delivery of a suicide gene can be used to reduce unexpected off-target toxicity.^49^

In conclusion, we present herein novel EPHB4-targeting CAR-T cells produced by PB transposon-based gene transfer for the clinical treatment of RMS and pediatric solid tumors. EPHB4-CAR-T cells exhibited sufficient CAR positivity, stable CAR expression, a favorable phenotype, and strong antitumor efficacy against EPHB4-positive tumors. EPHB4-CAR-T cell therapy shows potential as a treatment for RMS. A non-clinical study of EPHB4-CAR-T cell therapy targeting pediatric soft-tissue sarcoma is underway, and future clinical trials are proposed to be held in Japan.

## Materials and methods

### Blood donor, cell lines

PBMCs were obtained from normal healthy volunteers with informed consent. The study was approved by the Institutional Review Boards of the Kyoto Prefectural University of Medicine (approval nos. 2019-111 and 2019-112). Human RMS cell lines (Rh30, RD) were purchased from the American Type Culture Collection (Manassas, VA, USA), and Rh41 was kindly provided by Dr. Peter J. Houghton. The human osteosarcoma cell line, U2OS; the human breast cancer cell line, BT549; the human lymphoblastoid cell line, Raji; and HEK293 cells were purchased from the Japanese Cancer Research Resources Bank (JCRB) Cell Bank. All cells were maintained in Dulbecco’s modified Eagle’s medium (DMEM) (Nacalai Tesque, Kyoto, Japan), supplemented with 10% fetal bovine serum (Invitrogen, Carlsbad, CA, USA). Rh30-Luc was obtained by transfecting RediFect Red-Luc-Puromycin lentiviral particles (PerkinElmer, Waltham, MA, USA) on Rh30 cells. All cells were incubated in a humidified incubator under 5% CO_2_ at 37°C conditions. Human or murine EPHB4-expressing cells were obtained by transducing the human *EPHB4* (GenBank: NM_004444) or murine *Ephb4* (GenBank: NM_010144) gene together with the green fluorescent protein (*GFP*) gene into HEK293 cells via a PB transposon, following which the transduced cells were further purified using Sony SH800 cell sorter (Sony Biotechnologies, San Jose, CA, USA).

To produce human or murine EPHB4-expressing HEK293 cells, we transduced a pIRII-humanEPHB4-GFP or a pIRII-murineEPHB4-GFP plasmid, which contains *GFP* and human *EPHB4* (GenBank: NM_004444) or murine *Ephb4* (GenBank: NM_010144), respectively, into HEK293 cells, along with a PB transposase plasmid for stable expression using a MaxCyte ATX electroporator (MaxCyte, Gaithersburg, MD, USA). Transduced cells were expanded, and GFP-expressing cells were purified via flow cytometry using a Sony SH800S cell sorter (Sony Biotechnologies) to establish human or murine EPHB4-expressing HEK293 cells (293-humanEPHB4-GFP or 293-murneEPHB4-GFP), respectively. HEK293 cells expressing GFP were also generated for the EPHB4-negative control (293-GFP).

### EPHB4-CAR design

We generated a PB transposon-mediated transgene pIRII-EPHB4-CAR-28z, which enables transduced cells to express EPHB4-CAR. The antigen-recognition site of EPHB4-CAR was created using the extracellular domain of human EPHRIN B2 (GenBank: NP_004084) encoded by *EFNB2* (GenBank: NM_004093) and was cloned to the PB transposon vector by replacing the CD19 scFv portion of pIRII-CD19-CAR.^50^ The pIRII-tEPHB4-CD80-CD137L plasmid contains sequences encoding extracellular, transmembrane, and 20 amino acid intracellular portions of the EPHB4 protein (GenBank: NP_004435), following CD80 (GenBank: NP_005182) and CD137L (4-1BBL) (GenBank: NP_003802) with T2A and P2A self-cleaving sites, which enable each gene to be expressed independently. This deletion mutant EPHB4-CD80-CD137L sequence was artificially synthesized (Fasmac, Kanagawa, Japan) and cloned into a pIRII PB transposon vector (pIRII-tEPHB4-CD80-CD137L) (Figure S7). Likewise, the pIRII-tCD19-CD80-CD137L plasmid (Figure S7) was created by replacing the tEPHB4-CD80-CD137L portion of pIRII-tEPHB4-CD80-CD137L with truncated CD19 sequences encoding the extracellular, transmembrane, and 20 amino acids of the intracellular portion of the CD19 protein (GenBank: NP_001171569), CD80, and 4-1BBL with T2A and P2A self-cleaving sites.

### Gene transfer into T cells and expansion of transgenic T cells

The CAR transgene was transduced to PBMCs using the PB transposon system, as described previously.^50^ Briefly, the PB transposase plasmid (7.5 μg) and pIRII-EPHB4-CAR-28z (7.5 μg) or pIRII-CD19-CAR-28z (7.5 μg) were introduced into 4 × 10^7^ PBMCs using a MaxCyte ATX electroporator (MaxCyte), according to an optimized protocol (RTC 14-3) for introducing DNA plasmids into resting T cells. Concurrently, pIRII-tEPHB4-CD80-CD137L (4.5 μg) or pIRII-tCD19-CD80-CD137L (4.5 μg) was introduced into 1.5 × 10^7^ PBMCs via electroporation to serve as feeder cells. Following electroporation, CAR-T and feeder cells were cultured via a complete culture medium (CCM) consisting of ALyS705 medium (Cell Science & Technology Institute, Sendai, Japan), supplemented with 5% artificial serum (animal free; Cell Science & Technology Institute), interleukin (IL)-7 (10 ng/mL; Miltenyi Biotec, Auburn, CA, USA), and IL-15 (5 ng/mL; Miltenyi Biotec). Feeder cells were irradiated with ultraviolet light to inactivate them 24 h after electroporation and co-cultured with CAR-T cells in CCM for 14 days. These cells were transferred to chamber 1 of a MACS GMP Cell Differentiation Bag-100 (Miltenyi Biotec) on day 3 and expanded to chambers 2 and 3 on days 7 and 10, respectively. On day 14, the cells were harvested and used in subsequent examinations.

### Flow cytometry

EPHB4 expression in the tumor cell lines was determined via staining with a rat phycoerythrin (PE)-conjugated human EPHB4 antibody (R&D Systems, Minneapolis, MN, USA). In order to determine EPHB4-CAR expression, transduced T cells were stained with goat anti-human/mouse/rat Ephrin-B2 (R&D Systems) and stained with PE-conjugated anti-goat immunoglobulin G (IgG) antibody (R&D Systems). Allophycocyanin (APC)-conjugated anti-CD3 antibody, APC-conjugated anti-CD8 antibody, fluorescein isothiocyanate (FITC)-conjugated CD4 antibody, FITC-conjugated anti-CD45RA antibody, APC-conjugated anti-CCR7 antibody, PE-conjugated anti-human CD28 antibody, and PE-conjugated anti-human CD95 antibody (all from BioLegend, San Diego, CA, USA) were used to characterize CAR-T cell phenotypes. APC-conjugated anti-PD-1 antibody, APC-conjugated TIM3 antibody, and Alexa Fluor 647 anti-CD223 (LAG3) antibody were used as the exhaustion marker of CAR-T cells (all from BioLegend). CytotellRED 650 (AAT Bioquest, Sunnyvale, CA, USA) was used in a serial tumor challenge assay to assess the proliferation of EPHB4-CAR-T cells. APC-conjugated anti-human IgG Fc (BioLegend) was used to determine whether human recombinant EPHRIN B2-Fc (R&D Systems) binds with 293-human or -murine EPHB4-GFP cells. All flow cytometry data were acquired using a BD Accuri C6 Plus system (BD Biosciences, San Jose, CA, USA) and analyzed using the FlowJo Software v.10 package (Tree Star, Ashland, OR, USA).

### Cytokine production assay

To determine the response of target cells to EPHB4-CAR-T cells, the production levels of IL-1β, IL-2, IL-6, IL-12p70, tumor necrosis factor (TNF)-α, and IFN-γ were measured using a Cytometric Bead Array (CBA) Kit (BD Biosciences). Briefly, tumor cells were co-cultured with CAR-T cells at an E:T ratio of 1:1. Following 24 h of co-culture, cell culture supernatants were collected, and cytokine concentration was measured and analyzed. Data were acquired on a BD Accuri C6 Plus (BD Biosciences) and analyzed via FCAP Array v.3.0 (BD Biosciences).

### *In vitro* killing assay

To determine the killing effect of EPHB4-CAR-T cells on the target cells, RMS cell lines were co-cultured with EPHB4-CAR-T cells for 24 h at an E:T ratio of 1:1. The cell mixture was then collected and stained with PE-conjugated anti-insulin growth factor 1 receptor (IGF1R) antibody (BioLegend) and APC-conjugated anti-CD3 antibody (BioLegend) to identify RMS cells and T cells, respectively, and mixed with 50,000 CountBright Absolute Counting Beads (Thermo Fisher Scientific, Waltham, MA, USA). The number of live tumor cells (IGF1R-positive, CD3-negative cell fraction) was then determined via flow cytometry until the number of counting beads reached 5,000.

### Impedance-based tumor cell killing assay

An *in vitro* killing study was conducted using xCELLigence Real-Time Cell Analysis (RTCA) technology (ACEA Biosciences, San Diego, CA, USA). Briefly, after a background impedance measurement, 1 × 10^4^ tumor cells were seeded onto an E-plate 16. Approximately 24 h after seeding, 5 × 10^3^, 1 × 10^4^, or 2 × 10^4^ EPHB4-CAR-T cells were added for the purpose of conducting a co-culture experiment. In order to determine the immunomodulatory effect of P3F on tumor cells, siRNA was added to each tumor cell approximately 24 h after seeding, and the EPHB4-CAR-T cells were added to each cell approximately 48 h after seeding. The E-plate was placed on the RTCA single-plate (SP) Station located inside the incubator (5% CO_2_, 37°C) to continuously record impedance. Cell growth and adhesion were monitored every 15 min for approximately 150 h. Electrical impedance was measured and presented as the cell index (CI), and data were analyzed using software v.2.0 (ACEA Biosciences).

### Serial tumor challenge assay

We co-cultured 1 × 10^4^ Rh30 cells and 1 or 2 × 10^4^ EPHB4-CAR-T cells (E:T ratios of 1:1 or 2:1, respectively) on an E-plate 16. 3 days later, the EPHB4-CAR-T cells were collected and counted and then re-plated and reconstituted with fresh Rh30 cells at ratios of 1:1 or 2:1. Cell counting and re-plating were repeated every 3 days for a total of 3 iterations. The killing effect of the EPHB4-CAR-T cells was analyzed using xCELLigence RTCA. We also cultured Rh30 cells for 72 h in a 24-well plate and compared the normalized CI 72 h after the co-culture on the E-plate to determine the killing effect of EPHB4-CAR-T cells on Rh30 cells after co-culture.

### *In vivo* mouse xenograft studies

Female CB17.Cg-Prkdc^scid^Lyst^bg-J^/CrlCrlj mice (SCID beige mice, 4 weeks old) were purchased from Japan Oriental BioService (Kyoto, Japan). All experiments and procedures were approved by the Kyoto Prefectural University of Medicine Institutional Review Board (permit no. M30-140). Food and water were available *ad libitum*. The Rh30-Luc cell line, which constitutively expresses the luciferase gene, was used in the present study. We injected 2 × 10^6^ Rh30-Luc cells in 50 μL PBS into the dorsal areas of each mouse (N = 12, 6/group). After tumor engraftment was confirmed, 7 days post-transplantation, using an IVIS Lumina Series III System (PerkinElmer, Waltham, MA, USA), 1 × 10^7^ CD19-CAR-T cells or 1 × 10^7^ EPHB4-CAR-T cells were injected via the tail vein into RMS xenograft-bearing mice. The tumor volume was measured using an IVIS Imaging System. Briefly, mice were injected intraperitoneally (i.p.) with D-luciferin (150 mg/kg), 10 min before image acquisition, twice per week. Then the mice were anesthetized with 2% isoflurane, relocated to a heated stage in the chamber, and continuously exposed to 2% isoflurane for sustained sedation during imaging. Regions of interest (ROIs) on the displayed images were quantified in photons per second (ph/s) with Living Image v.2 (PerkinElmer). The mice were euthanized at predefined endpoints under conditions that met euthanasia criteria stipulated by the Center for Comparative Medicine at the Kyoto Prefectural University of Medicine.

### siRNA

Double-stranded RNA complementary to P3F was synthesized by targeting the fusion sites between exon 7 of *PAX3* and exon 2 of *FOXO1* (Table S1). We used siCON (Thermo Fisher Scientific) as a control for siPF. Then, siRNA transfections were carried out using Lipofectamine RNAiMAX (Thermo Fisher Scientific), according to the manufacturer’s recommended procedures. The concentration of siRNAs was diluted to 5 nM.

### Quantitative RT-PCR

Following transfection with siRNAs at indicated concentrations for 24 h, total RNA was extracted using a RNeasy Mini Kit (QIAGEN, Hilden, Germany) and transcribed into cDNA using a SuperScript First-Strand Synthesis System for RT-PCR (Invitrogen). Quantitative RT-PCR was conducted in a 7500 Real-Time PCR System (Applied Biosystems, Foster City, CA, USA) with TB Green (Takara Bio, Shiga, Japan). Cycle threshold (CT) values for target mRNAs and the gene encoding glucose-6-phosphate dehydrogenase (*GAPDH*) for each sample were calculated to quantify target mRNA levels, normalized to the constitutively expressed gene *GAPDH*. A normalized target value was then derived by subtracting the amount of target. The relevant primers are shown (Table S2).

### Western blot

Rh30 cells cultured for 15 min with or without 2 μg/mL clustered human recombinant EPHRIN B2-Fc and control human IgG (both from R&D Systems) were harvested and lysed with phosphatase and protease containing NP-40 buffer for protein purification. Total protein (30 μg) was separated by sodium dodecyl sulfate-polyacrylamide gel electrophoresis (SDS-PAGE), and proteins were transferred to polyvinylidene fluoride (PVDF) membranes (Bio-Rad, Hercules, CA, USA). After blocking with 4% bovine serum albumin, the membrane was incubated with primary antibodies against EPHB4, Erk1/2, phospho-Erk1/2, Akt, phospho-Akt, Crkl, phospho-Crkl, and β-actin (all from Cell Signaling Technologies, Danvers, MA, USA), according to the manufacturer’s instructions, following which the membrane was washed and incubated with horseradish peroxidase-conjugated secondary antibodies (Santa Cruz Biotechnology, Santa Cruz, CA, USA). The immunoreactive bands were visualized using an Amersham enhanced chemiluminescence (ECL) Prime Western Blot Detection Kit (GE Healthcare, Chicago, IL, USA).

### Human EPHRIN B2-Fc-binding assay

293-GFP, 293-humanEPHB4-GFP, and 293-murineEPHB4-GFP cells were incubated with 2 μg/mL of recombinant human Ephrin B2-Fc chimera protein (R&D Systems) for 30 min on ice. These cells were washed twice using Dulbecco’s PBS (D-PBS) and further stained with APC-conjugated anti IgG-Fc antibody (BioLegend) for 30 min on ice, following which cells bound to human Ephrin B2 chimera protein were detected by flow cytometry.

### Statistical analysis

The two-tailed Student’s t test was used to determine the statistical significance of differences between samples. All data are represented by the mean ± standard deviation. Statistical significance was set at p < 0.05. The log-rank test was used to compare survival curves obtained using the Kaplan-Meier method. Statistical analyses were performed on GraphPad Prism 7 software.

## Supplemental information

Document S1. Figures S1–S7 and Tables S1 and S2

Document S2. Article plus supplemental information

## References

[1] Porter D.L., Levine B.L., Kalos M., Bagg A., June C.H. (2011). Chimeric antigen receptor-modified T cells in chronic lymphoid leukemia. N. Engl. J. Med..

[2] Brentjens R.J., Davila M.L., Riviere I., Park J., Wang X., Cowell L.G., Bartido S., Stefanski J., Taylor C., Olszewska M. (2013). CD19-targeted T cells rapidly induce molecular remissions in adults with chemotherapy-refractory acute lymphoblastic leukemia. Sci. Transl. Med..

[3] Brentjens R.J., Rivière I., Park J.H., Davila M.L., Wang X., Stefanski J., Taylor C., Yeh R., Bartido S., Borquez-Ojeda O. (2011). Safety and persistence of adoptively transferred autologous CD19-targeted T cells in patients with relapsed or chemotherapy refractory B-cell leukemias. Blood.

[4] Kochenderfer J.N., Dudley M.E., Feldman S.A., Wilson W.H., Spaner D.E., Maric I., Stetler-Stevenson M., Phan G.Q., Hughes M.S., Sherry R.M. (2012). B-cell depletion and remissions of malignancy along with cytokine-associated toxicity in a clinical trial of anti-CD19 chimeric-antigen-receptor-transduced T cells. Blood.

[5] Ahmed N., Brawley V., Hegde M., Bielamowicz K., Kalra M., Landi D., Robertson C., Gray T.L., Diouf O., Wakefield A. (2017). HER2-Specific Chimeric Antigen Receptor-Modified Virus-Specific T Cells for Progressive Glioblastoma: A Phase 1 Dose-Escalation Trial. JAMA Oncol..

[6] Brown C.E., Badie B., Barish M.E., Weng L., Ostberg J.R., Chang W.C., Naranjo A., Starr R., Wagner J., Wright C. (2015). Bioactivity and Safety of IL13Rα2-Redirected Chimeric Antigen Receptor CD8+ T Cells in Patients with Recurrent Glioblastoma. Clin. Cancer Res..

[7] Louis C.U., Savoldo B., Dotti G., Pule M., Yvon E., Myers G.D., Rossig C., Russell H.V., Diouf O., Liu E. (2011). Antitumor activity and long-term fate of chimeric antigen receptor-positive T cells in patients with neuroblastoma. Blood.

[8] Park J.R., Digiusto D.L., Slovak M., Wright C., Naranjo A., Wagner J., Meechoovet H.B., Bautista C., Chang W.C., Ostberg J.R., Jensen M.C. (2007). Adoptive transfer of chimeric antigen receptor re-directed cytolytic T lymphocyte clones in patients with neuroblastoma. Mol. Ther..

[9] Majzner R.G., Mackall C.L. (2018). Tumor Antigen Escape from CAR T-cell Therapy. Cancer Discov..

[10] Zhang B.L., Qin D.Y., Mo Z.M., Li Y., Wei W., Wang Y.S., Wang W., Wei Y.Q. (2016). Hurdles of CAR-T cell-based cancer immunotherapy directed against solid tumors. Sci. China Life Sci..

[11] Zhang E., Gu J., Xu H. (2018). Prospects for chimeric antigen receptor-modified T cell therapy for solid tumors. Mol. Cancer.

[12] Ferguson B.D., Liu R., Rolle C.E., Tan Y.H., Krasnoperov V., Kanteti R., Tretiakova M.S., Cervantes G.M., Hasina R., Hseu R.D. (2013). The EphB4 receptor tyrosine kinase promotes lung cancer growth: a potential novel therapeutic target. PLoS ONE.

[13] Kumar S.R., Scehnet J.S., Ley E.J., Singh J., Krasnoperov V., Liu R., Manchanda P.K., Ladner R.D., Hawes D., Weaver F.A. (2009). Preferential induction of EphB4 over EphB2 and its implication in colorectal cancer progression. Cancer Res..

[14] Kumar S.R., Singh J., Xia G., Krasnoperov V., Hassanieh L., Ley E.J., Scehnet J., Kumar N.G., Hawes D., Press M.F. (2006). Receptor tyrosine kinase EphB4 is a survival factor in breast cancer. Am. J. Pathol..

[15] Hasina R., Mollberg N., Kawada I., Mutreja K., Kanade G., Yala S., Surati M., Liu R., Li X., Zhou Y. (2013). Critical role for the receptor tyrosine kinase EPHB4 in esophageal cancers. Cancer Res..

[16] Huang X., Yamada Y., Kidoya H., Naito H., Nagahama Y., Kong L., Katoh S.Y., Li W.L., Ueno M., Takakura N. (2007). EphB4 overexpression in B16 melanoma cells affects arterial-venous patterning in tumor angiogenesis. Cancer Res..

[17] Randolph M.E., Cleary M.M., Bajwa Z., Svalina M.N., Young M.C., Mansoor A., Kaur P., Bult C.J., Goros M.W., Michalek J.E. (2017). EphB4/EphrinB2 therapeutics in Rhabdomyosarcoma. PLoS ONE.

[18] Egea J., Klein R. (2007). Bidirectional Eph-ephrin signaling during axon guidance. Trends Cell Biol..

[19] Janes P.W., Nievergall E., Lackmann M. (2012). Concepts and consequences of Eph receptor clustering. Semin. Cell Dev. Biol..

[20] Chrencik J.E., Brooun A., Kraus M.L., Recht M.I., Kolatkar A.R., Han G.W., Seifert J.M., Widmer H., Auer M., Kuhn P. (2006). Structural and biophysical characterization of the EphB4∗ephrinB2 protein-protein interaction and receptor specificity. J. Biol. Chem..

[21] Su S.A., Xie Y., Zhang Y., Xi Y., Cheng J., Xiang M. (2019). Essential roles of EphrinB2 in mammalian heart: from development to diseases. Cell Commun. Signal..

[22] Chen Y., Zhang H., Zhang Y. (2019). Targeting receptor tyrosine kinase EphB4 in cancer therapy. Semin. Cancer Biol..

[23] Noren N.K., Lu M., Freeman A.L., Koolpe M., Pasquale E.B. (2004). Interplay between EphB4 on tumor cells and vascular ephrin-B2 regulates tumor growth. Proc. Natl. Acad. Sci. USA.

[24] Aslam M.I., Abraham J., Mansoor A., Druker B.J., Tyner J.W., Keller C. (2014). PDGFRβ reverses EphB4 signaling in alveolar rhabdomyosarcoma. Proc. Natl. Acad. Sci. USA.

[25] Mahnke Y.D., Brodie T.M., Sallusto F., Roederer M., Lugli E. (2013). The who’s who of T-cell differentiation: human memory T-cell subsets. Eur. J. Immunol..

[26] Xu Y., Zhang M., Ramos C.A., Durett A., Liu E., Dakhova O., Liu H., Creighton C.J., Gee A.P., Heslop H.E. (2014). Closely related T-memory stem cells correlate with in vivo expansion of CAR.CD19-T cells and are preserved by IL-7 and IL-15. Blood.

[27] Kagoya Y., Nakatsugawa M., Ochi T., Cen Y., Guo T., Anczurowski M., Saso K., Butler M.O., Hirano N. (2017). Transient stimulation expands superior antitumor T cells for adoptive therapy. JCI Insight.

[28] Fraietta J.A., Lacey S.F., Orlando E.J., Pruteanu-Malinici I., Gohil M., Lundh S., Boesteanu A.C., Wang Y., O’Connor R.S., Hwang W.T. (2018). Determinants of response and resistance to CD19 chimeric antigen receptor (CAR) T cell therapy of chronic lymphocytic leukemia. Nat. Med..

[29] Tanaka M., Tashiro H., Omer B., Lapteva N., Ando J., Ngo M., Mehta B., Dotti G., Kinchington P.R., Leen A.M. (2017). Vaccination Targeting Native Receptors to Enhance the Function and Proliferation of Chimeric Antigen Receptor (CAR)-Modified T Cells. Clin. Cancer Res..

[30] Linardic C.M. (2008). PAX3-FOXO1 fusion gene in rhabdomyosarcoma. Cancer Lett..

[31] Kikuchi K., Tsuchiya K., Otabe O., Gotoh T., Tamura S., Katsumi Y., Yagyu S., Tsubai-Shimizu S., Miyachi M., Iehara T., Hosoi H. (2008). Effects of PAX3-FKHR on malignant phenotypes in alveolar rhabdomyosarcoma. Biochem. Biophys. Res. Commun..

[32] Nabarro S., Himoudi N., Papanastasiou A., Gilmour K., Gibson S., Sebire N., Thrasher A., Blundell M.P., Hubank M., Canderan G., Anderson J. (2005). Coordinated oncogenic transformation and inhibition of host immune responses by the PAX3-FKHR fusion oncoprotein. J. Exp. Med..

[33] Xia G., Kumar S.R., Masood R., Koss M., Templeman C., Quinn D., Zhu S., Reddy R., Krasnoperov V., Gill P.S. (2005). Up-regulation of EphB4 in mesothelioma and its biological significance. Clin. Cancer Res..

[34] Xia G., Kumar S.R., Masood R., Zhu S., Reddy R., Krasnoperov V., Quinn D.I., Henshall S.M., Sutherland R.L., Pinski J.K. (2005). EphB4 expression and biological significance in prostate cancer. Cancer Res..

[35] Sinha U.K., Kundra A., Scalia P., Smith D.L., Parsa B., Masood R., Gill P.S. (2003). Expression of EphB4 in head and neck squamous cell carcinoma. Ear Nose Throat J..

[36] Chrencik J.E., Brooun A., Recht M.I., Nicola G., Davis L.K., Abagyan R., Widmer H., Pasquale E.B., Kuhn P. (2007). Three-dimensional structure of the EphB2 receptor in complex with an antagonistic peptide reveals a novel mode of inhibition. J. Biol. Chem..

[37] Dai D., Huang Q., Nussinov R., Ma B. (2014). Promiscuous and specific recognition among ephrins and Eph receptors. Biochim. Biophys. Acta.

[38] Zhao C., Irie N., Takada Y., Shimoda K., Miyamoto T., Nishiwaki T., Suda T., Matsuo K. (2006). Bidirectional ephrinB2-EphB4 signaling controls bone homeostasis. Cell Metab..

[39] Rutkowski R., Mertens-Walker I., Lisle J.E., Herington A.C., Stephenson S.A. (2012). Evidence for a dual function of EphB4 as tumor promoter and suppressor regulated by the absence or presence of the ephrin-B2 ligand. Int. J. Cancer.

[40] Kertesz N., Krasnoperov V., Reddy R., Leshanski L., Kumar S.R., Zozulya S., Gill P.S. (2006). The soluble extracellular domain of EphB4 (sEphB4) antagonizes EphB4-EphrinB2 interaction, modulates angiogenesis, and inhibits tumor growth. Blood.

[41] Martiny-Baron G., Korff T., Schaffner F., Esser N., Eggstein S., Marmé D., Augustin H.G. (2004). Inhibition of tumor growth and angiogenesis by soluble EphB4. Neoplasia.

[42] Djokovic D., Trindade A., Gigante J., Badenes M., Silva L., Liu R., Li X., Gong M., Krasnoperov V., Gill P.S., Duarte A. (2010). Combination of Dll4/Notch and Ephrin-B2/EphB4 targeted therapy is highly effective in disrupting tumor angiogenesis. BMC Cancer.

[43] Noren N.K., Foos G., Hauser C.A., Pasquale E.B. (2006). The EphB4 receptor suppresses breast cancer cell tumorigenicity through an Abl-Crk pathway. Nat. Cell Biol..

[44] Hu B., Ren J., Luo Y., Keith B., Young R.M., Scholler J., Zhao Y., June C.H. (2017). Augmentation of Antitumor Immunity by Human and Mouse CAR T Cells Secreting IL-18. Cell Rep..

[45] Pegram H.J., Lee J.C., Hayman E.G., Imperato G.H., Tedder T.F., Sadelain M., Brentjens R.J. (2012). Tumor-targeted T cells modified to secrete IL-12 eradicate systemic tumors without need for prior conditioning. Blood.

[46] Craddock J.A., Lu A., Bear A., Pule M., Brenner M.K., Rooney C.M., Foster A.E. (2010). Enhanced tumor trafficking of GD2 chimeric antigen receptor T cells by expression of the chemokine receptor CCR2b. J. Immunother..

[47] Condomines M., Arnason J., Benjamin R., Gunset G., Plotkin J., Sadelain M. (2015). Tumor-Targeted Human T Cells Expressing CD28-Based Chimeric Antigen Receptors Circumvent CTLA-4 Inhibition. PLoS ONE.

[48] Prosser M.E., Brown C.E., Shami A.F., Forman S.J., Jensen M.C. (2012). Tumor PD-L1 co-stimulates primary human CD8(+) cytotoxic T cells modified to express a PD1:CD28 chimeric receptor. Mol. Immunol..

[49] Diaconu I., Ballard B., Zhang M., Chen Y., West J., Dotti G., Savoldo B. (2017). Inducible Caspase-9 Selectively Modulates the Toxicities of CD19-Specific Chimeric Antigen Receptor-Modified T Cells. Mol. Ther..

[50] Morita D., Nishio N., Saito S., Tanaka M., Kawashima N., Okuno Y., Suzuki S., Matsuda K., Maeda Y., Wilson M.H. (2017). Enhanced Expression of Anti-CD19 Chimeric Antigen Receptor in *piggyBac* Transposon-Engineered T Cells. Mol. Ther. Methods Clin. Dev..

