# Supplementary material for: Development of non-viral, ligand-dependent, EPHB4-specific chimeric antigen receptor T cells for treatment of rhabdomyosarcoma
Source: Mol Ther Oncolytics. 2021 Mar 5;20:646–58. doi: 10.1016/j.omto.2021.03.001 (PMC7985479; doi:10.1016/j.omto.2021.03.001)
Supplement: Document S1. Figures S1–S7 and Tables S1 and S2 [file mmc1.pdf]

OMTO, Volume *20*

Supplemental information

Development of non-viral, ligand-dependent, EPHB4-specific chimeric antigen receptor T cells for treatment of rhabdomyosarcoma

Hiroshi Kubo, Shigeki Yagyu, Kayoko Nakamura, Kumiko Yamashima, Akimasa Tomida, Ken Kikuchi, Tomoko Iehara, Yozo Nakazawa, and Hajime Hosoi

Figure S1. Kubo H et al.

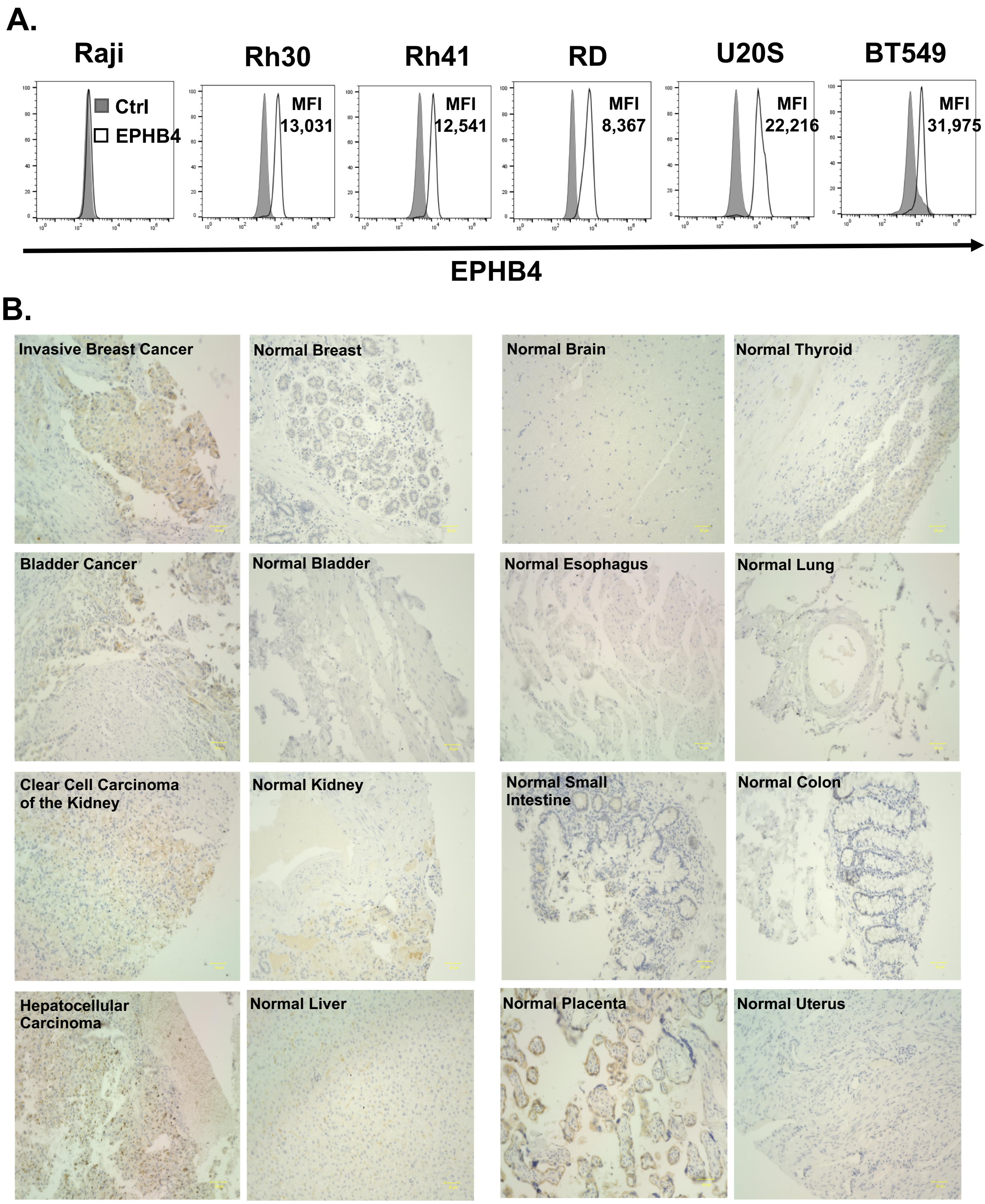

Figure S1

A. EPHB4 expressed in rhabdomyosarcoma (RMS) (Rh30, Rh41, RD), osteosarcoma (U2OS), and breast cancer (BT549) cell lines. EPHB4 was barely expressed in the lymphoblastoid cell line (Raji) (negative control). **B.** EPHB4 expression in tumor tissue and normal tissue examined via immunohistochemistry. Scale bar: 50 µm.

Figure S2. Kubo H et al.

A.

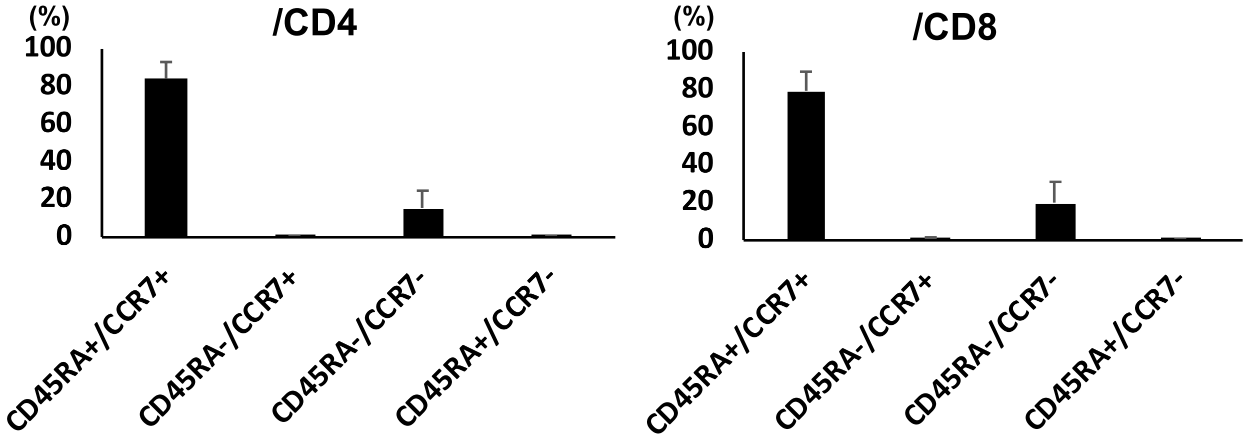

B.

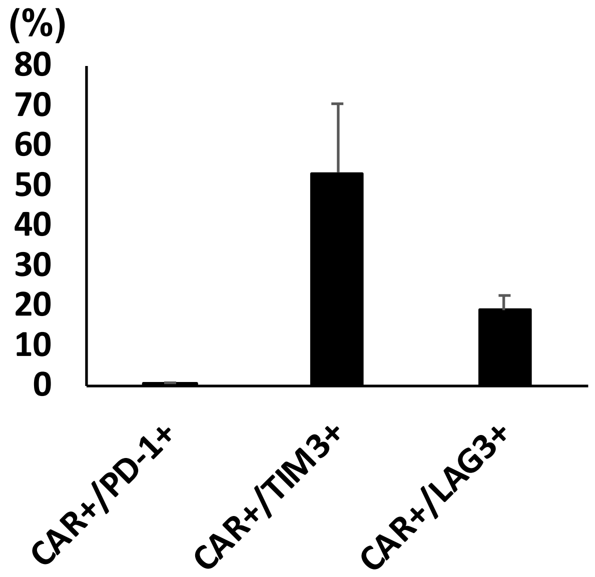

Figure S2

A. The phenotypes on EPHB4-CAR-T cells in CD4 positive (left) and CD8 positive (right) subpopulation were assessed via flow cytometry. **B.** The exhaustion markers on CAR positive of EPHB4-CAR-T cells. Data are represented by the means ± SD (N = 5).

Figure S3. Kubo H et al.

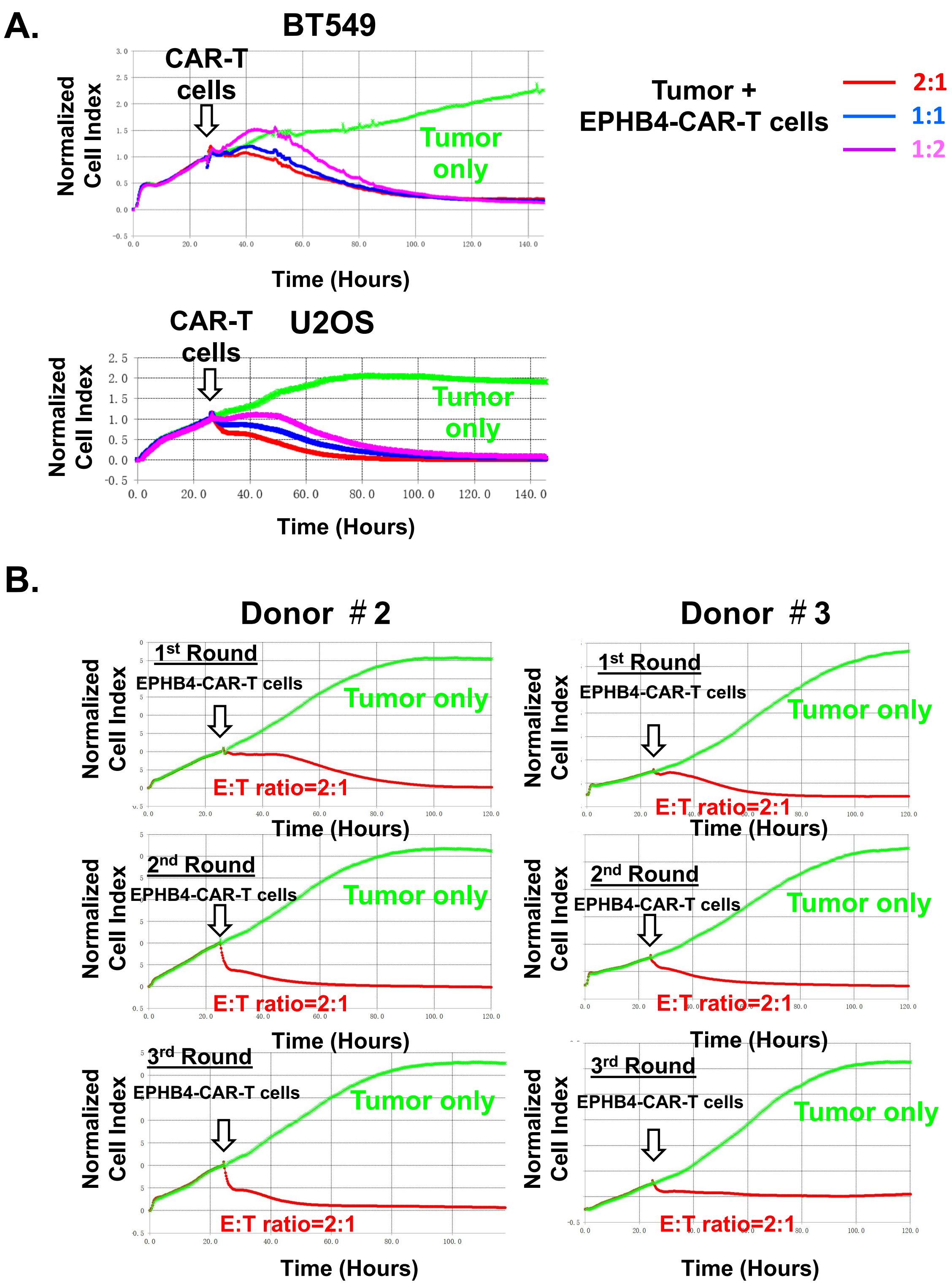

Figure S3

A. BT549 and U2OS were co-cultured with EPHB4-CAR-T cells at E:T ratios of 2:2, 1:1, and 1:2. **B.** Serial tumor challenge assay in another two donors (Donor#2, Donor#3) in addition to the data shown in Figure 2B.

Figure S4. Kubo H et al.

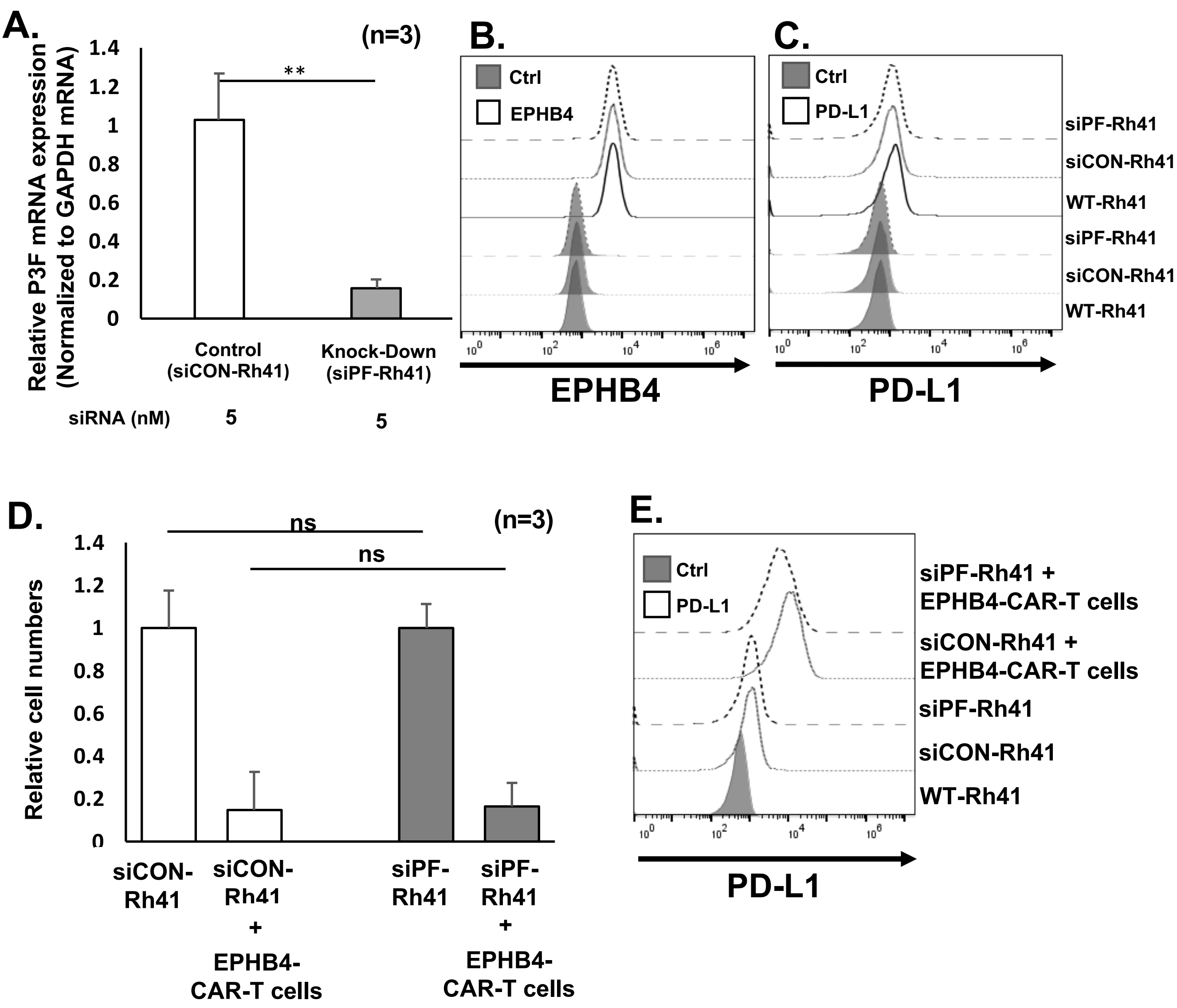

Figure S4

In relation to Figure 3, we have validated the data using another translocation positive RMS cell line, Rh41. **A.** After transfection with siRNAs against P3F (siPF) into P3F-positive Rh41 cells for 24 h, the knocked-down efficacy of siPF against P3F was assessed by quantitative reverse-transcription polymerase chain reaction (qRT-PCR). ** = P < 0.01. **B.** P3F did not affect EPHB4 and **C.** PD-L1 assessed by flow cytometry. **D.** A comparison of the antitumor effect in siCON-Rh41 and siPF-Rh41 assessed by flow cytometry. The antitumor effect of the EPHB4-CAR-T cells on Rh41 cells was evaluated according to the ratio of survival of Rh41 cells alone to the survival of Rh41 cells cocultured with EPHB4-CAR-T cells. Mean ± SD from 3 different experiments are shown; ns = not significant **E.** PD-L1 expression between siCON-Rh41 and siPF-Rh41 treated with EPHB4-CAR-T cells was evaluated via flow cytometry.

Figure S5. Kubo H et al.

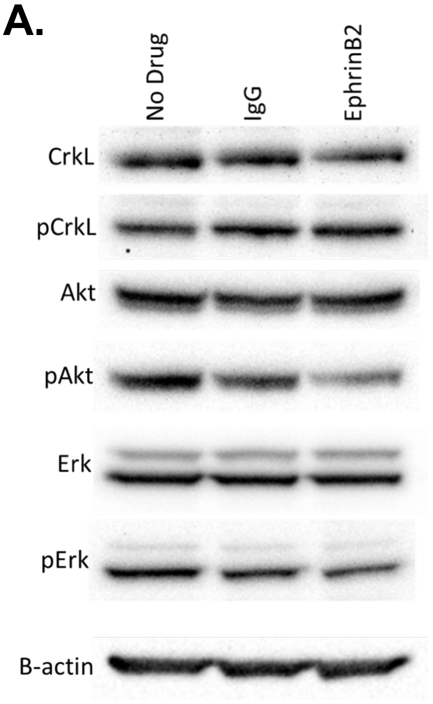

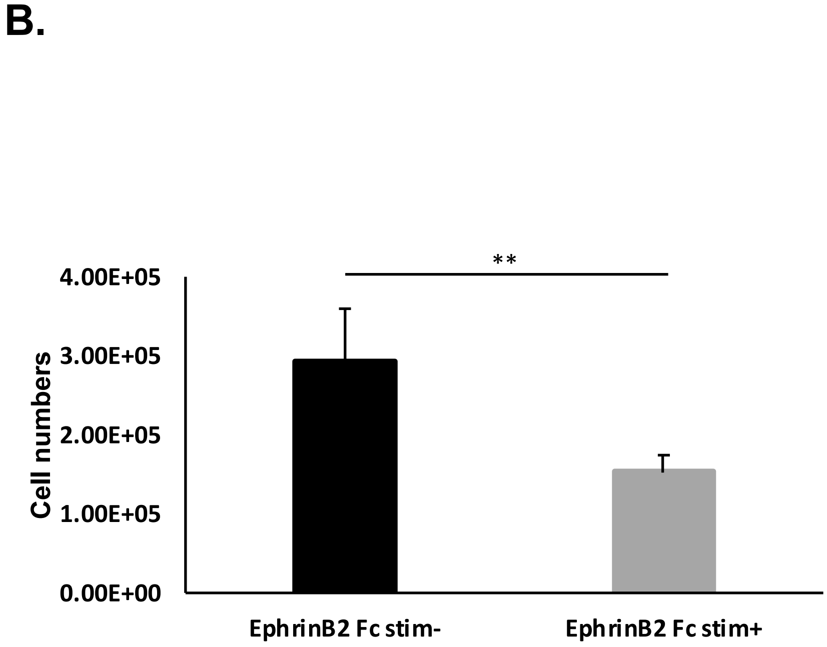

Figure S5

A. Protein expression levels of Crkl, phospho-Crkl (pCrkl), Akt, phospho-Akt (pAkt), Erk1/2, and phospho-Erk1/2 (pErk) were determined via western blot analysis using β-actin as a control. Rh30 cells were cultured with 2 μg/mL clustered human recombinant EPHRIN B2-Fc or human IgG for 15 min. **B.** Rh30 cells cultured on EphrinB2-Fc coated plate for 72 h and the number of live tumor cells were measured via flow cytometry; ** = $P < 0.01$

Figure S6. Kubo H et al.

A.

```
EPHB4_HUMAN  LEETLLNTKLETADLKWVTFPQVDGQWEELSGLDEEQHSVRTYEVCDVQRAPGQAHWLRT
EPHB4_MOUSE  LEETLLNTKLETADLKWVTYPQAEGQWEELSGLDEEQHSVRTYEVCDMKRPGGQAHWLRT

EPHB4_HUMAN  GWVPRRGAVHVYATLRFTMLECLSLPRAGRSCKETFTVFYYESDADTATALTPAWMENPY
EPHB4_MOUSE  GWVPRRGAVHVYATIRFTMMECLSLPRASRSCKETFTVFYYESEADTATAHTPAWMENPY

EPHB4_HUMAN  IKVDTVAAEHLTRKRPGAEATGKVNVKTLRLGPLSKAGFYLAFQDQGACMALLSLHLFYK
EPHB4_MOUSE  IKVDTVAAEHLTRKRPGAEATGKVNIKTLRLGPLSKAGFYLAFQDQGACMALLSLHLFYK

EPHB4_HUMAN  KCAQLTVNLTRFPETVPRELVVPVAGSCVVDAVPAPGPSPSLYCREDGQWAEQPVTGCSC
EPHB4_MOUSE  KCSWLITNLTYFPETVPRELVVPVAGSCVANAVPTANPSPSLYCREDGQWAEQQVTGCSC

EPHB4_HUMAN  APGFEAAEGNTKCRACAQGTFKPLSGEGSCQPCPANSHSNTIGSAVCQCRVGYFRARTDP
EPHB4_MOUSE  APGYEAAESNKVCRACGQGTFKPQIGDESCLPCPANSHSNNIGSPVCLCRIGYYRARSDP

EPHB4_HUMAN  RGAPCTTPPSAPRSVVSRLNGSSLHLEWSAPLESGGREDLTYALRCRECRPGGSCAPCGG
EPHB4_MOUSE  RSSPCTTPPSAPRSVVHHLNGSTLRLEWSAPLESGGREDLTYAVRCRECRPGGSCLPCGG

EPHB4_HUMAN  DLTFDPGPRDLVEPWVVVRGLRPDFTYTFEVTALNGVSSLATGPVPFEPVNVTTDREVPP
EPHB4_MOUSE  DMTFDPGPRDLVEPWVAIRGLRPDVTYTFEVAALNGVSTLATGPPPFEPVNVTTDREVPP

EPHB4_HUMAN  AVSDIRVTRSSPSSLSLAWAVPRAPSGAVLDYEVKYHEKGAEGPSSVRFLKTSENRAELR
EPHB4_MOUSE  AVSDIRVTRSSPSSLILSWAIPRAPSGAVLDYEVKYHEKGAEGPSSVRFLKTSENRAELR

EPHB4_HUMAN  GLKRGASYLVQVRARSEAGYGPFGQEHHSQTQLDESEGWREQLA
EPHB4_MOUSE  GLKRGASYLVQVRARSEAGYGPFGQEHHSQTQLDESESWREQLA
```

B.

```
EFNB2_HUMAN  IVLEPIYWNSSNSKFLPGQGLVLYPQIGDKLDIICPKVDSKTVGQYEYYKVYMVDKDQAD
EFNB2_MOUSE  IVLEPIYWNSSNSKFLPGQGLVLYPQIGDKLDIICPKVDSKTVGQYEYYKVYMVDKDQAD

EFNB2_HUMAN  RCTIKKENTPLLNCAKPDQDIKFTIKFQEFSPNLWGLEFQKNKDYYIISTSNGSLEGLDN
EFNB2_MOUSE  RCTIKKENTPLLNCARPDQDVKFTIKFQEFSPNLWGLEFQKNKDYYIISTSNGSLEGLDN

EFNB2_HUMAN  QEGGVCQTRAMKILMKVGQDASSAGSTRNKDPTRRPELEAGTNGRSSTTSPFVKPNPGSS
EFNB2_MOUSE  QEGGVCQTRAMKILMKVGQDASSAGSARNHGPTRRPELEAGTNGRSSTTSPFVKPNPGSS

EFNB2_HUMAN  TDGNSAGHSGNNILGSEVALFA
EFNB2_MOUSE  TDGNSAGHSGNNLLGSEVALFA
```

Figure S6

A. The amino acid sequences of human and murine EPHB4 receptor regions contain a conserved 186-amino-acid N-terminal ligand-binding domain (EPH LBD, highlighted in green). **B.** The amino acid sequences of human and mouse EPHRIN B2 (EFNB2) regions contain a conserved 137-amino-acid N-terminal receptor-binding domain (Ephrin RBD, highlighted in magenta)

Figure S7. Kubo H et al.

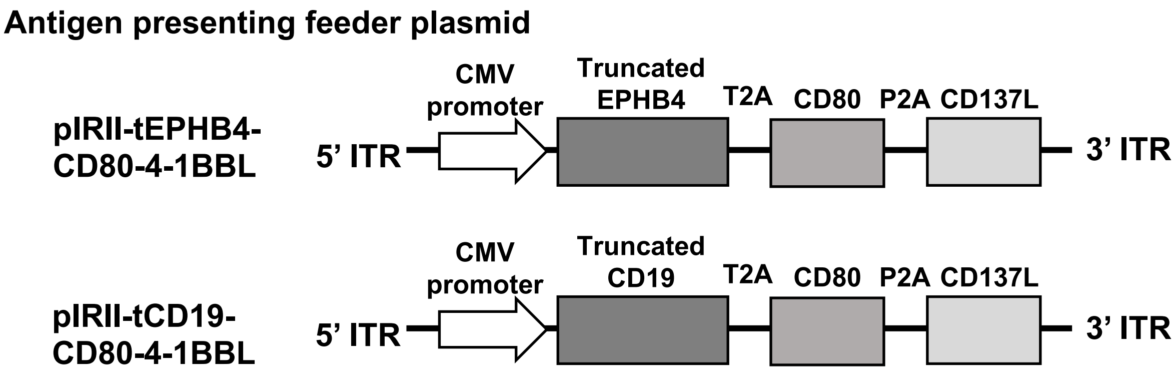

Figure S7

Schematic of antigen presenting feeder plasmid for EPHB4-CAR and of CD19-CAR.

Supplemental Table 1

siRNA and primer sequences

siRNA name		Sequence 5′ to 3′
siPF	s	CCUCUCACCUCAGAAUUCATT
siPF	as	UGAAUUCUGAGGUGAGAGGTT
siCON	s	CUACUAUACCGAUACUCCCTT
siCON	as	GGGAGUAUCGGUAUAGUAGTT

Supplemental Table 2

Forward and reverse primer sequences

Target gene		Sequence 5′ to 3′
PAX3-FOXO1	F	TCCAACCCCATGAACCCC
	R	GCCATTTGGAAAACTGTGATCC
GAPDH	F	GCACCGTCAAGGCTGAGAAC
	R	ATGGTGGTGAAGACGCCAGT
